# Correction: López-Paterna et al. Quality of Life, Perceived Social Support, and Treatment Adherence Among Methadone Maintenance Program Users: An Observational Cross-Sectional Study. *Healthcare* 2025, *13*, 1849

**DOI:** 10.3390/healthcare13243297

**Published:** 2025-12-16

**Authors:** Pedro López-Paterna, Ismail Erahmouni-Bensliman, Raquel Sánchez-Ruano, Ricardo Rodríguez-Barrientos, Milagros Rico-Blázquez

**Affiliations:** 1 La Velada Healthcare Centre, Área de Gestión Sanitaria Campo de Gibraltar Este, Servicio Andaluz de Salud, 11300 La Línea de la Concepció, Spain; 2 Algeciras Norte Healthcare Centre, Área de Gestión Sanitaria Campo de Gibraltar Este, Servicio Andaluz de Salud, 11205 Algeciras, Spain; 3 Research Unit, Primary Care Assistance Management, Madrid Health Service, 28035 Madrid, Spain; 4 Research Network on Chronicity, Primary Care and Health Promotion-RICAPPS-(RICORS), Instituto de Salud Carlos III (ISCIII), 28003 Madrid, Spain; 5 Gregorio Marañon Health Research Institute, Madrid Health Service, 28007 Madrid, Spain; 6 Nursing Department, Faculty of Nursing, Physiotherapy and Podiatry, Complutense University of Madrid, 28040 Madrid, Spain

There was an error in the original publication [1]. The use of the MMAS-8 Scale requires a license. The requirements in the license to use this scale are to add a statement to the footnote of the first table or figure which presents the MMAS-8, and Acknowledgment Section in the manuscript, and cite three specific references.

Corrections have been made to the caption of Figure 6 and the Acknowledgments Section.

**Figure 6.** Treatment adherence of the participants (MMAS-8). The MMAS-8 Scale, content, name, and trademarks are protected by US copyright and trademark laws. Permission for use of the scale and its coding is required. A license agreement is available from MMAR, LLC., www.moriskyscale.com.

**Acknowledgments:** We thank all the nurses, security personnel, coordinators, managers, and center directors for facilitating the field work and accompanying us during recruitment. During the preparation of this manuscript, the authors used ChatGPT 4 to improve the readability and language of the manuscript. This project has a license (number 1784102025) for the use of the MMAS-8 Scale. The MMAS-8 Scale, content, name, and trademarks are protected by US copyright and trademark laws. Permission for use of the scale and its coding is required. A license agreement is available from MMAR, LLC., www.moriskyscale.com. The authors have reviewed and edited the output and take full responsibility for the content of this publication.

The following specified references have been added in Section 2. Materials and Methods, 2.2. Variables and Information Collection, paragraph 1:

38. Krousel-Wood, M.; Islam, T.; Webber, L.S.; Re, R.N.; Morisky, D.E.; Muntner, P. New Medication Adherence Scale Versus Pharmacy Fill Rates in Seniors With Hypertension. *Am. J. Manag. Care* **2009**, *15*, 59−66. PMID 19146365; PMCID: PMC27285932.
39. Berlowitz, D.R.; Foy, C.G.; Kazis, L.E.; Bolin, L.; Lonroy, L.B.; Fitzpatrick, P.; Gure, T.R.; Kimmel, P.L.; Kirscner, K.; Morisky, D.E.; et al. Impact of Intensive Blood Pressure Therapy on Patient-Reported Outcomes: Outcomes Results From the SPRINT Study. *N. Engl. J. Med.* **2017**, *377*, 733−744.
40. Bress, A.P.; Bellows, B.K.; King, J.B.; Hess, R.; Beddhu, S.; Zhang, Z.; Berlowitz, D.R.; Conroy, M.B.; Fine, L.; Oparil, S.; et al. Cost-Effectiveness of Intensive Versus Standard Blood-Pressure Control. *N. Engl. J. Med.* **2017**, *377*, 745−755.

And the full paragraph should read:

Data were collected through self-administered questionnaires in health centers between July 2024 and March 2025. Three scales that have been validated in Spain [33–35] were used to measure the main outcome variables: (I) the World Health Organization Quality of Life Instrument—Short Version (WHOQoL-BREF) is a 26-item instrument that measures quality of life across four domains: physical health, psychological well-being, social relationships, and the environment. Each domain is scored on a scale from 0 to 100, with higher scores indicating better perceived quality of life [36]. (II) The Morisky Medication Adherence Scale (MMAS-8) is an 8-item scale that measures self-reported medication adherence. Total scores range from 0 to 8, with higher scores indicating better adherence, which is categorized as follows: high adherence (score = 8), medium adherence (score of 6–8), and low adherence (score < 6) [37–40]. (III) The Duke–UNC Functional Social Support Questionnaire (DUKE-UNC-11) is an 11-item scale that evaluates the perception of functional social support. Total scores range from 11 to 55, and lower scores indicate lower perceived social support. In Spain, a score below 32 is considered indicative of low social support [41].

With this correction, the order of some references has been adjusted accordingly. The authors state that the scientific conclusions are unaffected. This correction was approved by the Academic Editor. The original publication has also been updated.
